# End-of-life decision-making across cancer types: results from a nationwide retrospective survey among treating physicians

**DOI:** 10.1038/s41416-018-0070-5

**Published:** 2018-03-29

**Authors:** Mariëtte N. Verkissen, Dirk Houttekier, Joachim Cohen, Rik Schots, Kenneth Chambaere, Luc Deliens

**Affiliations:** 1 End-of-Life Care Research Group, Vrije Universiteit Brussel (VUB) and Ghent University, Laarbeeklaan 103, 1090 Brussels, Belgium; 2 Department of Clinical Haematology, Universitair Ziekenhuis Brussel, Laarbeeklaan 101, 1090 Brussels, Belgium

**Keywords:** Palliative care, Health policy

## Abstract

**Background:**

The treatment of advanced cancer often involves potentially life-shortening end-of-life decisions (ELDs). This study aimed to examine the prevalence and characteristics of ELDs in different cancer types.

**Methods:**

A nationwide death certificate study was conducted based on a large random sample of all deaths in Flanders, Belgium, between 1 January and 30 June 2013. All cancer deaths were selected (*n* = 2392). Attending physicians were sent a questionnaire about ELDs and the preceding decision-making process.

**Results:**

The response rate was 58.3%. Across cancer types, a non-treatment decision occurred in 7.6–14.0%, intensified pain and symptom alleviation in 37.5–41.7%, euthanasia or physician-assisted suicide in 8.7–12.6%, and life shortening without explicit patient request in 1.0–2.4%. ELD prevalence did not differ significantly by cancer type. Reasons for ELDs were most frequently patient’s physical suffering and lack of prospect of improvement. ‘Anticipated further suffering’ and ‘unbearable situation for relatives’ were reasons more often reported in haematological cancer than in other cancer types. Patient, family, and caregiver involvement in decision-making did not differ across cancer types.

**Conclusions:**

Euthanasia or physician-assisted suicide rates were relatively high in all cancer types. Neither the prevalence of ELDs nor characteristics of the decision-making process differed substantially between cancer types. This indicates a uniform approach to end-of-life care, including palliative care, across oncological settings.

## Introduction

Many people with advanced cancer face difficult treatment decisions at the end of life. In responding to these decisions, patients need to balance the realistic possibilities of prolonging life by continuing treatment or starting new treatments on the one hand, against comfort and quality of life on the other hand.^1^ As a result, it can happen that decisions are made that may potentially hasten the patient’s death. This can be a foreseen but intended consequence or an intended outcome. These end-of-life decisions with a possible or certain life-shortening effect (ELDs), on which this article particularly focuses, include non-treatment decisions (withholding or withdrawing of potentially life-sustaining treatment), intensifying pain or symptom alleviation (increasing pain or symptom relief by administering high-dose drugs while taking into account the possibility or certainty that this could hasten death), and administering drugs with the explicit intention of hastening death (euthanasia, physician-assisted suicide, or life shortening without the individual’s explicit request). Although euthanasia and physician-assisted suicide are still illegal in most countries, a growing number of countries now have legislation allowing one or both of these practices (Belgium, the Netherlands, Luxembourg, Colombia, Canada, five US states and Switzerland).^2^

Previous research has shown that ELDs, specifically intensified symptom alleviation, euthanasia, and physician-assisted suicide, are more frequent in people dying from cancer than in people dying from other conditions.^3–6^ Possible explanations may be that symptom burden is higher in cancer than in other diseases^7,8^ and that palliative care services—specializing in symptom management—are more readily available or accessible.^9–12^ Moreover, there is a trend in cancer care toward earlier diagnosis and open doctor-patient communication about the prognosis,^13^ which may lead to more ELDs.

Previous studies have described ELDs in cancer patients.^3,5,14,15^ Unfortunately, none have examined the prevalence of ELDs and characteristics of the decision-making process in different cancer types, although this could be valuable for several reasons. Equity of care aims to ensure that the quality of end-of-life care provided does not differ by characteristics unrelated to a patient’s needs.^16^ We know, however, from previous studies that people with different types of cancer may receive different types and intensity of end-of-life care, which may for example be related to differences in symptoms or trajectories of functional decline at the end of life.^17–20^ Patients with haematological cancer in particular tend to have poorer access to palliative care services, are more likely to be admitted to an intensive care unit and to receive aggressive end-of-life care, and are less likely to die at home than those with solid malignancies.^20–28^ Such variations across cancer types, which could point to potential inequities in the provision end-of-life care to people with advanced cancer, may also exist regarding the occurrence of ELDs and characteristics of decision-making, e.g., the extent to which physicians involve patients and their families in this process.

Understanding the relationship between cancer type and end-of-life decision-making could help inform policy and practice about whether tailoring terminal care to specific cancer diagnoses is needed. The aim of this study is to examine the prevalence of ELDs and the characteristics of the decision-making process in people dying from different cancer types in 2013 in Flanders, Belgium, a country where euthanasia has been legal under specific conditions since 2002. The specific research questions are (1) what is the prevalence of different types of ELDs across cancer types; (2) what are physicians’ reasons for making ELDs across cancer types; (3) to what extent are patients and families involved in the decision-making process across cancer types; and (4) to what extent are professional caregivers involved in the decision-making process across cancer types?

## Patients and methods

### Study design

A death certificate study was conducted, based on a representative sample of deaths in Flanders, Belgium. The Flemish Agency for Care and Health (FACH) selected a random sample of all deaths of Belgian residents (aged 1 year and above) between 1 January and 30 June 2013, stratified for the likelihood of an ELD being made. Larger samples were taken for strata in which the cause of death, as indicated on the death certificate, made an ELD more likely.^29^ This resulted in a total sample of 6871. For this analysis, all deaths in the database with an underlying cause of cancer were selected (*n* = 2669).

Certifying physicians were sent a four-page questionnaire via standard mail within 2 months of the death concerning medical decisions made at the end of life, the decision-making process, and the care provided. They were requested to complete the questionnaire by consulting the patient’s medical file. If the certifying physician was not the treating physician, the questionnaire was passed on to the treating physician. A lawyer acting as intermediary between responding physicians, researchers, and the FACH ensured that completed questionnaires could never be linked to a particular patient or physician. After data collection, a one-page questionnaire was mailed to all non-respondents asking their reasons for not participating. The mailing and anonymity procedures were approved by the Ethical Review Board of the University Hospital of the Vrije Universiteit Brussel, the Belgian National Disciplinary Board of Physicians, and the Belgian Privacy Commission. The study design, sampling, and mailing procedure are described in detail elsewhere.^29,30^

### Questionnaire

The questionnaire, tested thoroughly by a panel of physicians, was largely identical to those of previous studies in Belgium, the Netherlands and other European countries, the first of which had been developed for the 1990 Dutch survey on ELDs.^31^ In a validation study, the classification of ELDs that was based on the responses given on the postal survey matched the classification that was based on the responses given in face-to-face interviews.^32^ The questionnaire first asked whether death had been sudden and unexpected and whether the attending physician’s first contact with the patient had been after death. If the answer to both these questions was no—hence end-of-life decision-making before death was not precluded—they were then asked whether they had (1) withheld or withdrawn life-prolonging medical treatment (e.g., chemotherapy, artificial provision of nutrition and hydration, provision of antibiotics, and mechanical ventilation) taking into account or explicitly intending the hastening of death (non-treatment decision); (2) intensified the alleviation of pain and/or other symptoms with drugs with the possibility of hastening death (intensified alleviation of pain and symptoms); or (3) administered, supplied or prescribed drugs with the explicit intention of hastening death. If in the latter case someone other than the patient at the patient’s explicit request had administered the drugs, the act was classified as euthanasia; if drugs had been prescribed or supplied and self-administered, the act was classified as physician-assisted suicide. If there had been no explicit request from the patient, the act was classified as life abbreviation without explicit patient request. For patients for whom more than one ELD was made, the act with the most explicit life-shortening intention was regarded as the most important ELD. When two decisions with similar life-shortening intention were made, administering drugs was regarded as prevailing over withholding or withdrawing treatment as the most important ELD. Questions then followed about the reasons for the most important ELD and about the decision-making process (the involvement of the patient, family, and professional caregivers in making the decision).

Data on sex, age, place, and underlying cause of death were available from the individually linked death certificate information. The underlying cause of death variable was coded according to ICD-10. People dying from cancer were identified according to ICD-10 codes C00 to C97: gastrointestinal (C15-26); respiratory (C30-39, C45-49); genitourinary (C51-58, C60-63, C64-68); breast (C50); head and neck (C00-C14); haematological (C81-96); and ‘other’ for other types of cancer. These included bone and articular cartilage (C40-41); skin (C43-44); eye, brain, and central nervous system (C69-72); thyroid and endocrine glands (C73-75); ill-defined, secondary and unspecified sites (C76-80); and independent (primary) multiple sites (C97).

### Statistical analysis

The subset of cancer-related deaths was weighted to be representative of all cancer deaths in the first half of 2013 in terms of age, sex, marital status, province of death, cause of death, and place of death (adjustments were only needed for place of death). After this weighting procedure there were no significant differences between the sampled cancer deaths and all cancer deaths on any of these variables.

Descriptive statistics on prevalence of ELDs, types of ELDs, reasons for the ELD, and the involvement of the patient, family, and professional caregivers in the decision-making process are presented for different cancer types. Results for euthanasia and physician-assisted suicide were combined because the latter only occurred in five cases. Deaths from head and neck cancer (*n* = 41) were combined with the ‘other’ category (*n* = 131) because of its small group size. Bivariate cross-tabulations and multivariable logistic regression models were calculated to compare patients dying from different cancer types, with statistical significance set at *p* < .05. Bivariate differences between cancer types were calculated using Pearson *χ*^2^-tests. Multivariable regression models incorporated the most important confounders to determine the independent effect of cancer type on ELDs and the preceding decision-making process. All statistical analyses were performed with complex samples functions in IBM SPSS Statistics version 23.0.

## Results

Questionnaires were returned for 1394 of 2669 deaths. Non-response questionnaires revealed that responding was impossible in 277 cases, e.g., because the physician did not have access to the medical file or the patient could not be identified. Therefore, the response rate was 58.3% (1394 of 2392 cases).

### Case characteristics

Patients dying from different cancer types differed significantly in age at death, sex, living situation, marital status, and place of death (Table 1).

**Table 1:** Characteristics of patients dying from cancer in 2013 in Flanders, Belgium (*n* = 1394)

Cancer type								
	All cancer deaths (*n* = 1394)	Gastrointestinal (*n* = 417)	Respiratory (*n* = 334)	Genitourinary (*n* = 252)	Breast (*n* = 113)	Haematological (*n* = 106)	Other^a^ (*n* = 172)	
Characteristics	Weighted %^b^							*P*-value^c^
*Age at death, years*								
1–64	24.1	21.4	31.1	10.0	31.5	16.6	37.2	**<.001**
65–79	41.0	42.4	45.9	41.9	42.3	29.6	33.4	
≥ 80	34.9	36.2	22.9	48.1	26.2	53.8	29.4	
*Sex*								
Male	57.1	57.9	71.0	60.3	0.0	62.7	58.3	**<.001**
Female	42.9	42.1	29.0	39.7	100.0	37.3	41.7	
*Living situation*								
Alone	22.2	21.1	21.3	25.1	22.6	26.3	18.9	**.025**
In household with others	66.2	68.9	70.8	60.9	60.4	54.9	70.2	
Institution	11.6	9.9	7.9	14.0	16.9	18.8	10.9	
*Marital status*								
Single	8.2	9.7	8.6	4.1	6.1	9.7	10.4	**.001**
Married	55.1	55.3	60.1	56.8	49.5	43.8	53.9	
Widowed	26.9	27.6	17.5	30.3	37.6	37.8	24.1	
Divorced	9.8	7.4	13.8	8.8	6.9	8.7	11.6	
*Place of death*								
At home	30.6	29.7	36.0	29.9	26.3	19.2	33.7	**.002**
Hospital	58.0	59.2	57.3	54.2	56.9	66.3	56.7	
Care home	11.5	11.2	6.7	15.8	16.8	14.5	9.7	
*Treating physician*								
Clinical specialist	55.4	54.5	56.9	51.8	53.1	65.6	54.6	.310
General practitioner	44.6	45.5	43.1	48.2	46.9	34.4	45.4	

Percentages are column percentages

Not included in table and analyses: ‘other’ category for living situation *n* = 5 (0.4% of all cancer deaths), missing data for living situation *n* = 10 (0.7% of all cancer deaths), ‘unknown’ category for marital status *n* = 4 (0.3% of all cancer deaths), ‘other’ category for place of death *n* = 34 (2.4% of all cancer deaths), missing data for place of death *n* = 1 (0.1% of all non-sudden cancer deaths), ‘other’ category for treating physician *n* = 61 (4.4% of all cancer deaths), missing data for treating physician *n* = 18 (1.3% of all cancer deaths). ^a^‘Other’ category includes head and neck; bone and articular cartilage; skin; eye, brain and central nervous system; thyroid and endocrine glands; ill-defined, secondary and unspecified sites; independent (primary) multiple sites

^b^Percentages are weighted for the disproportionate stratification and differences in the distribution of mortality characteristics between the response sample and all patient deaths

^c^Bivariate differences between patients with different cancer types are calculated using Pearson’s *χ*^2^-tests. Bold denotes significance at *p* < .05

### Prevalence of end-of-life decisions across cancer types

Of all cancer deaths, 22.6% were judged to be sudden and unexpected, and 66.1% were preceded by an end-of-life decision, ranging from 60.9% in breast cancer to 69.4% in respiratory cancer (Table 2). A non-treatment decision was made in 7.6% (breast) to 14.0% (respiratory), intensified alleviation of pain and symptoms in 37.5% (haematological) to 44.0% (other). Euthanasia or physician-assisted suicide occurred in 8.7% (genitourinary) to 12.6% (respiratory), and life abbreviation without the explicit request from the patient in 1.0% (respiratory) to 3.4% (genitourinary).

**Table 2:** Prevalence of end-of-life decisions in patients dying from cancer in 2013 in Flanders, Belgium (*n* = 1394)

	ELD made or not			Type of ELD made			
	Sudden and unexpected death, no ELD made	Non-sudden death, no ELD made	Non-sudden death, at least one ELD made	Non-treatment decision	Intensified alleviation of pain or symptoms	Euthanasia or physician-assisted suicide^a^	Life abbreviation without explicit patient request
Cancer type	Weighted %^b^			Weighted %^b^			
All cancer deaths (*n* = 1394)	22.6	11.3	66.1	12.1	41.7	10.4	1.8
Gastrointestinal (*n* = 417)	23.8	10.8	65.5	12.3	41.4	10.5	1.3
Respiratory (*n* = 334)	20.0	10.6	69.4	14.0	41.8	12.6	1.0
Genitourinary (*n* = 252)	24.2	8.8	67.1	12.2	42.8	8.7	3.4
Breast (*n* = 113)	21.5	17.6	60.9	7.6	41.2	10.2	1.9
Haematological (*n* = 106)	22.1	16.6	61.3	12.3	37.5	9.0	2.4
Other^c^ (*n* = 172)	23.8	9.7	66.5	11.0	44.0	9.7	1.8
*P*-value^d^	.868	.132	.590	.724	.947	.750	.465

Percentages are row percentages

*ELD* end-of-life decision with possible or certain life-shortening effect

^a^Five of 211 patients were cases of physician-assisted suicide

^b^Percentages are weighted for the disproportionate stratification and differences in the distribution of mortality characteristics between the response sample and all patient deaths

^c^‘Other’ category includes head and neck; bone and articular cartilage; skin; eye, brain, and central nervous system; thyroid and endocrine glands; ill-defined, secondary, and unspecified sites; independent (primary) multiple sites

^d^Bivariate differences between patients with different cancer types calculated using Pearson’s *χ*^2^-tests

The proportion of deaths in which an ELD was made did not differ significantly between cancer types. Also, no significant differences in the occurrence of specific ELDs were found. These associations remained non-significant in multivariable regression models adjusting for age, sex, marital status, and place of death (results not shown).

### Reasons for end-of-life decisions across cancer types

The reason most often reported by physicians for the most important ELD was physical suffering, ranging from 71.0% in haematological cancer to 87.0% in breast cancer (Table 3). Other important reasons included a lack of prospect of improvement (60.4% other, to 78.6% haematological), the wish of the patient (40.0% other, to 46.2% respiratory), anticipated further suffering (26.0% other, to 53.8% haematological), not to prolong life needlessly (29.7% breast, to 43.5% haematological), and expected poor quality of life (23.0% other, to 46.1% haematological). In approximately a quarter of cases, reasons for making the ELD were related to the wishes of the family.

**Table 3:** Reasons for end-of-life decisions in patients dying from cancer (*n* = 963)

Reasons for ELD										
	Physical suffering	No prospect of improvement	Wish of the patient	Expected further suffering	Life not to be prolonged needlessly	Expected poor quality of life	Wish of the family	Loss-of-dignity	Mental suffering	Unbearable situation for relatives
Cancer type	Weighted %^a^									
All cancer deaths for which at least one ELD was made (*n* = 963)	76.8	66.9	43.0	38.6	38.4	36.5	28.0	21.9	21.5	12.4
Gastrointestinal (*n* = 291)	71.8	65.1	41.9	35.8	32.1	37.4	23.8	21.6	19.6	10.5
Respiratory (*n* = 233)	78.9	70.0	46.2	43.4	42.7	40.9	29.5	18.6	22.6	10.8
Genitourinary (*n* = 178)	79.9	62.7	40.4	34.3	43.4	35.6	29.2	25.1	20.7	10.9
Breast (*n* = 71)	87.0	72.4	45.4	48.5	29.7	31.5	30.3	25.5	32.6	10.4
Haematological (*n* = 68)	71.0	78.6	45.9	53.8	43.5	46.1	25.5	24.8	23.9	23.4
Other^b^ (*n* = 122)	77.5	60.4	40.0	26.0	39.2	23.0	33.4	20.5	17.3	16.9
*P*-value^c^	.115	.130	.853	**.003**	.098	**.034**	.559	.723	.322	.054

Concerns the most important ELD. More than one reason was possible for each case

Percentages are row percentages *ELD* end-of-life decision with possible or certain life-shortening effect

Not included in table and analyses: ‘other’ category reasons for ELDs *n* = 15 (1.6% of all cancer deaths for which at least one ELD was made), missing data for reasons for ELDs* n* = 77 (8.0% of all cancer deaths for which at least one ELD was made)

^a^Percentages are weighted for the disproportionate stratification and differences in the distribution of mortality characteristics between the response sample and all patient deaths

^b^‘Other’ category includes head and neck; bone and articular cartilage; skin; eye, brain, and central nervous system; thyroid and endocrine glands; ill-defined, secondary and unspecified sites; independent (primary) multiple sites

^c^Bivariate differences between patients with different cancer types calculated using Pearson’s *χ*^2^-tests. Bold denotes significance at *p* < .05

No significant differences between cancer types were found in the reasons for ELDs, except for ‘expected further suffering’ (*p* = .003), and ‘expected poor quality of life’ (*p* = .034). In multivariable regression models adjusting for age, sex, marital status, place of death, and type of ELD, only the association between cancer type and ‘expected further suffering’ as a reason for the ELD remained (*p* = .006); this reason was more often reported in haematological cancer than in gastrointestinal, genitourinary, and other types of cancer. The association between cancer type and ‘unbearable situation for relatives’ became statistically significant after adjustment for confounders (*p* = .025); this reason was more often reported in haematological compared with gastrointestinal, breast, respiratory, and genitourinary cancer (results not shown).

### Involvement of patients and families in the decision-making process across cancer types

At the time the ELD was made, physicians found 66.2% of all cancer patients for whom at least one ELD was made to have had capacity for decision-making (Table 4). The ELD was discussed with 81.7% of patients judged to have capacity, ranging from 71.6% in genitourinary to 92.3% in haematological cancer. The ELD was made in response to an explicit patient request in 71.7% (gastrointestinal) to 82.3% (haematological) of cases where the patient was deemed to have capacity. For those lacking capacity, a written advance directive was present in 1.4% (genitourinary) to 14.4% (other) of cases, and the ELD was discussed with the family in 59.2% (other) to 74.9% (breast).

**Table 4:** Involvement of patients dying from cancer and their families in the end-of-life decision-making process (*n* = 963)

Involvement of patient and family in the decision-making process								
	All cancer deaths for which at least one ELD was made		All cancer deaths for which at least one ELD was made and patient had capacity			All cancer deaths for which at least one ELD was made and patient lacked capacity		
		Patient had capacity		Decision discussed with patient with capacity	Decision made in response to explicit request by patient with capacity		Patient without capacity had written advance directive (euthanasia or other)	Decision discussed with family of patient without capacity
Cancer type	*n*	Weighted %^a^	*n*	Weighted %^a^		*n*	Weighted %^a^	
All cancer deaths	963	66.2	613	81.7	74.2	272	5.6	64.3
Gastrointestinal	291	65.2	184	82.1	71.7	82	5.7	68.5
Respiratory	233	69.5	158	83.5	74.6	61	5.6	61.0
Genitourinary	178	63.1	109	71.6	73.6	56	1.4	62.5
Breast	71	69.9	44	86.9	77.1	18	5.9	74.9
Haematological	68	61.8	42	92.3	82.3	21	3.1	61.1
Other^b^	122	67.1	76	81.2	73.7	34	14.4	59.2
*P*-value^c^		.781		.069	.867		.179	.845

Concerns the most important ELD. More than one response was possible for each case *ELD* end-of-life decision with possible or certain life-shortening effect

Percentages are row percentages

Not included in table and analyses: missing data for patient had capacity *n* = 78 (8.1% of all cancer deaths for which at least one ELD was made), missing data for decision discussed with patient with capacity *n* = 2 (0.3% of all cancer deaths for which at least one ELD was made and patient had capacity), missing data for decision made in response to explicit request by patient with capacity *n* = 13 (2.1% of all cancer deaths for which at least one ELD was made and patient had capacity), missing data for decision discussed with family of patient without capacity *n* = 3 (1.1% of all cancer deaths for which at least one ELD was made and patient lacked capacity)

^a^Percentages are weighted for the disproportionate stratification and differences in the distribution of mortality characteristics between the response sample and all patient deaths

^b^‘Other’ category includes head and neck; bone and articular cartilage; skin; eye, brain, and central nervous system; thyroid and endocrine glands; ill-defined, secondary and unspecified sites; independent (primary) multiple sites

^c^Bivariate differences between patients with different cancer types calculated using Pearson’s *χ*^2^-tests

The capacity of the patient to make decisions was not related to cancer type. For those with capacity, the proportion of cases in which the ELD was discussed with them and the proportion in which the ELD was made in response to an explicit request from them did not differ by cancer type. For those without capacity, neither the presence of a written advance directive nor the decision being discussed with the family could be related to cancer type. All these associations remained non-significant in multivariable regression (results not shown).

### Involvement of professional caregivers in the decision-making process across cancer types

ELDs were discussed with a colleague physician in 38.3% (breast) to 57.8% (haematological) of all cases for which at least one ELD was made (Table 5). A palliative care specialist was consulted in 30.7% (haematological) to 44.7% (other), and nursing staff in 35.3% (breast) to 47.9% (other).

**Table 5:** Involvement of professional caregivers of patients dying with cancer in the end-of-life decision-making process (*n* = 963)

Involvement of professional caregivers in the decision-making process			
	Decision discussed with colleague physician(s)	Decision discussed with palliative care specialist	Decision discussed with nursing staff
Cancer type	Weighted %^a^		
All cancer deaths for which at least one ELD was made (*n* = 963)	50.9	37.0	43.3
Gastrointestinal (*n* = 291)	49.1	38.8	44.6
Respiratory (*n* = 233)	53.3	33.6	43.1
Genitourinary (*n* = 178)	53.1	38.4	41.0
Breast (*n* = 71)	38.3	32.8	35.3
Haematological (*n* = 68)	57.8	30.7	44.7
Other^b^ (*n* = 122)	49.8	44.6	47.9
*P*-value^c^	.340	.409	.741

Concerns the most important ELD. More than one response was possible for each case *ELD* end-of-life decision with possible or certain life-shortening effect

Percentages are row percentages

Not included in table and analyses: missing data for involvement of professional caregivers in the decision-making process *n* = 76 (7.9% of all cancer deaths for which at least one ELD was made)

^a^Percentages are weighted for the disproportionate stratification and differences in the distribution of mortality characteristics between the response sample and all patient deaths

^b^‘Other’ category includes head and neck; bone and articular cartilage; skin; eye, brain, and central nervous system; thyroid and endocrine glands; ill-defined, secondary and unspecified sites; independent (primary) multiple sites

^c^Bivariate differences between patients with different cancer types calculated using Pearson’s *χ*^2^-tests

The involvement of professional caregivers in the decision-making process was not related to cancer type, and remained unchanged in multivariable regression (results not shown).

## Discussion

End-of-life decisions were found to be equally common in all cancer types, with particularly high euthanasia or physician-assisted suicide rates (8.7–12.6% across cancer types). Our study did not find significant differences between different cancer types regarding the prevalence of ELDs and the involvement of patients, family, and professional caregivers in the decision-making process; ‘expected further suffering’ and ‘unbearable situation for relatives’ were reasons more often reported in those dying from haematological cancer than from other cancer types.

This is the first study estimating and comparing the occurrence of ELDs in different cancer types. An important strength is that it was based on a large random sample of death certificates. The population-based approach allows for international as well as national comparative research. Furthermore, the 58.3% response rate achieved is satisfactory, considering that average response rates among physicians to mailed surveys have been reported to be 54–61% in previous studies.^33–35^ This enabled us to analyse ELDs and characteristics of the preceding decision-making process in detail by cancer diagnosis. The findings of this study need also to be considered in light of its limitations. Despite the adequate response rate, we cannot fully exclude some degree of non-response bias, nor can we exclude the possibility of social desirability bias, especially an under-reporting of ELDs that may be considered contentious. Information was gathered from the physician’s perspective only, thereby excluding the perspectives of patients, their families and other professional caregivers. Finally, although physicians were encouraged to complete the questionnaire by consulting the medical file as much as possible, recall bias might have affected the results.

Our findings demonstrate that the prevalence of ELDs is equally high in all cancer types, suggesting that patients and oncologists see medical practices with a potential or certain life-shortening effect as acceptable end-of-life options. More than one in 10 deaths (10.4%) were from euthanasia or physician-assisted suicide, which is a considerable increase on the 5.6% of all cancer deaths recorded in 2007^6^ and substantially higher than the general rate of 4.6% recorded in 2013.^30^ An important finding of our study is that this high rate of assisted dying is consistently noticeable in all cancer groups (8.7–12.6%), and that it does not differ significantly between cancer types. Taking into account that the number of euthanasia requests is higher than the number eventually carried out, this indicates that in Belgium assisted dying has clearly become a part of medical practice in the care of cancer patients and that the various disciplines of oncology need to be trained in dealing with euthanasia requests.

The administration of drugs with the explicit intention to hasten death (life-shortening acts) without the explicit request from the patient occurred in 1.8% (1.0–3.4%) of the cancer deaths during the studied period. These acts have been central in debates on physician-assisted dying as they are often seen as indicators for the development of a ‘slippery slope’—the fear that even if legislation for euthanasia sets out strict criteria and safeguards, it will lead inevitably to undesirable practices.^36,37^ To date, no clear evidence has been found to support this fear. In Belgium and the Netherlands, where euthanasia has been legal for many years, the proportion of deaths in which life-ending drugs were used without explicit patient request has not risen since the legalisation.^38–40^ Previous studies have also identified important differences between cases of euthanasia and life-shortening acts without explicit patient request in terms of the drugs used.^41–44^ An analysis of cases of life-shortening acts without explicit patient request revealed that these acts mainly involved opioids, which are seldom used in euthanasia, and which were administered in doses that were not higher than needed for pain and symptom management. The practice of using life-ending drugs without explicit patient request may thus in reality be more similar to intensified pain alleviation with a ‘double effect’ than to non-voluntary termination of life.^42,44^ This, together with other studies showing that physicians tend to overestimate the life-shortening effect of opioids,^45–47^ may indicate a need for education of clinicians aimed at correcting misperceptions about opioid use.

Our data revealed no difference in ELD rates in patients dying from the five most common cancers. Although symptoms and access to palliative care services have been found to differ depending on cancer type,^17–19^ this seems not to be the case for decision-making at the end of life. A possible explanation for this may be that universal protocols, education and training of care practices across oncological settings or specialisms have resulted in a procedural, structured and uniform approach to providing care toward the end of life in cancer patients, including palliative care.

Regarding reasons for ELDs, it is not surprising that the patient’s physical suffering and the lack of prospects for improvement were mentioned most frequently by physicians, given the significant burden of symptoms that cancer patients can experience and taking into account that people with cancer have a more predictable dying trajectory compared to those with other chronic, terminal illnesses such as heart failure and chronic obstructive pulmonary disease.^13^ The frequency with which the reasons ‘expected further suffering’ and ‘unbearable situation for relatives’ were reported differed by cancer type; they were most often reported in haematological cancers. The spectrum of haematological cancer is very broad, with chronic diseases and low treatment-related toxicity (e.g., chronic leukaemias, low-grade myelodysplasias, chronic myeloproliferative neoplasms, indolent lymphomas) on the one hand, and rapidly evolving and lethal cancers (e.g., acute leukaemias, aggressive lymphomas, and multiple myelomas) on the other hand. In the latter group life expectancy is short, especially in the elderly population which happens to be the highest proportion included in this study (53.8% of patients with haematological cancer were ≥80 years). In this population, and taking into account the aggressive nature of these cancers, as well as the poor therapeutic options, it seems logical that ELDs are more frequently inspired by avoiding further suffering and the unbearability of the situation for close relatives.

Although the majority of patients with decision-making capacity were involved in the decision-making process preceding the ELD, decision-making took place without the patient’s input in almost 20% of cases. Oncologists face several barriers which may hamper effective communication with patients and families about end-of-life issues, such as personal discomfort with death and dying, diffusion of responsibility among colleagues, and lack of training in this area.^48^ Nevertheless, according to the ethical principle of patient autonomy, all possibly life-shortening decisions should be discussed with the patient, unless he or she has explicitly said otherwise.^49^ Also noteworthy is the low rate of written advance directives (<6%) and the suboptimal involvement of family members that we found in patients lacking decision-making capacity (the ELD was discussed with the family in <70% of cases in all cancer groups except for breast cancer). Advance care planning, which ideally includes, though is not limited to, the appointment of a proxy decision-maker and the completion of an advance directive, can be worthwhile for cancer patients when planning for the possibility of losing decision-making capacity.^50,51^

In conclusion, our results show high occurrence rates of ELDs in all cancer types. Neither the prevalence of ELDs nor the characteristics of the preceding decision-making process were found to differ greatly by cancer type, indicating that end-of-life care and palliative care are provided uniformly across cancer settings. Although our findings suggest that the decision-making process preceding an ELD is often inclusive, there is still room for improvement in encouraging the involvement of the person who is dying, as well as those close to them in decision-making toward the end of life. Patients with incurable cancer and their families may benefit from the timely initiation of discussions about future treatment and end-of-life care preferences, especially those with cancers that have a higher potential of rapid deterioration, such as haematological malignancies. Therefore, embedding advance care planning into usual oncology clinical practice is crucial. Health services and policy-makers need to develop strategies, guidelines, and procedures for implementation of appropriate advance care planning programs, and provide health care facilities with the necessary education and resources.

### Availability of data and material

Individual data cannot be made fully available due to data protection and privacy restrictions that were made under contract with the Flemish Agency for Care and Health, who collected the data. These restrictions prohibit the research group from sharing the collected data with others to prevent study participants from being identified. Aggregated data can be requested from the Flemish Agency (anne.kongs@wvg.vlaanderen.be) after requesting permission from the Privacy Commission (joris.ballet@ksz-bcss.fgov.be).
